# Efficacy of AI Chats to Determine an Emergency: A Comparison Between OpenAI’s ChatGPT, Google Bard, and Microsoft Bing AI Chat

**DOI:** 10.7759/cureus.45473

**Published:** 2023-09-18

**Authors:** Gabriel Zúñiga Salazar, Diego Zúñiga, Carlos L Vindel, Ana M Yoong, Sofia Hincapie, Ana B Zúñiga, Paula Zúñiga, Erin Salazar, Byron Zúñiga

**Affiliations:** 1 Facultad de Ciencias Médicas, Universidad Católica de Santiago de Guayaquil, Guayaquil, ECU; 2 Neurology, Hospital Luis Vernaza, Guayaquil, ECU; 3 Nephrology, Hospital Luis Vernaza, Guayaquil, ECU

**Keywords:** ai & robotics in healthcare, emergency department, emergency room, emergency, microsoft bing, google bard, chatgpt, chatbot, artificial intelligence

## Abstract

Background

The escalating overload and saturation of emergency services, primarily caused by non-urgent cases overwhelming the system, have spurred a critical necessity for innovative solutions that can effectively differentiate genuine emergencies from situations that could be managed through alternative means, such as using AI chatbots. This study aims to evaluate and compare the accuracy in differentiating between a medical emergency and a non-emergency of three of the most popular AI chatbots at the moment.

Methods

In this study, patient questions from the online forum r/AskDocs on Reddit were collected to determine whether their clinical cases were emergencies. A total of 176 questions were reviewed by the authors, with 75 deemed emergencies and 101 non-emergencies. These questions were then posed to AI chatbots, including ChatGPT, Google Bard, and Microsoft Bing AI, with their responses evaluated against each other and the authors’ responses. A criteria-based system categorized the AI chatbot answers as “yes,” “no,” or “cannot determine.” The performance of each AI chatbot was compared in both emergency and non-emergency cases, and statistical analysis was conducted to assess the significance of differences in their performance.

Results

In general, AI chatbots considered around 12-15% more cases to be an emergency than reviewers, while they considered a very low number of cases as non-emergency compared to reviewers (around 35% fewer cases). Google Bard detected the most true emergency cases (87%) and true non-emergency cases (36%). However, no real difference in performance between the three AI chatbots was found in detecting true emergencies (p-value = 0.35) and non-emergency cases (p-value = 0.16).

Conclusions

These AI systems require further refinement to identify emergency situations accurately, but they could potentially be an innovative tool for emergency care and improving patient outcomes. The integration of AI chatbots like ChatGPT, Google Bard, and Microsoft Bing Chat offers a promising avenue to mitigate ED strain and enhance emergency management.

## Introduction

A chatbot is a software program designed to imitate human conversation by means of text or voice interactions, typically online [1]. With diverse applications spanning across industries, these bots facilitate interactions, optimize processes, and enrich user experiences across various fields, making them valuable assets for both businesses and individuals [2]. Furthermore, chatbots are already actively used in the healthcare sector [3]. According to a study, medical professionals in the United States perceive chatbots as particularly useful for tasks like scheduling doctor appointments, locating health clinics, or offering medication information [4].

Modern chatbots are artificial intelligence (AI) systems that can engage in natural language conversations, mirroring human conversational behavior [5]. These technologies often incorporate components of deep learning and natural language processing. Recently, this domain has garnered considerable attention due to the popularity of OpenAI’s ChatGPT, along with alternatives like Microsoft’s Bing Chat (utilizing OpenAI’s GPT-4) and Google’s Bard.

On the other hand, the escalating overload and saturation of emergency services, primarily caused by non-urgent cases overwhelming the system, have spurred a critical necessity for innovative solutions that can effectively differentiate genuine emergencies from situations that could be managed through alternative means [6,7].

In this study, we aim to evaluate the integration of AI-powered chat systems as a promising strategy to ease the pressure on emergency services by efficiently assessing the urgency of situations and determining the need to seek emergency resources, thus ultimately contributing to more effective and resource-efficient emergency management systems.

## Materials and methods

In this cross-sectional study, we collected patients’ questions posted to an online social media forum, Reddit’s community r/AskDocs, which data has been used before for research purposes [8,9].

In July 2023, questions or clinical cases were searched in the subreddit r/AskDocs, in which patients asked about the following: (1) the need to go to the emergency room or (2) if their clinical case can be considered an emergency. In the subreddit search bar, the following keywords were input to search for these questions: “emergency,” “emergency room,” “emergency department,” “ER,” and “ED.” Only questions that asked “should I go to the emergency?” or “is this considered an emergency?” or similar, were considered.

Formal consent wasn’t required as the data came from public sources without personal details. We summarized quotes from posts to protect patients’ privacy and used the complete quotes to generate chatbot responses.

A total of 176 questions were obtained, and they were subjected to a review process by the authors, who each reviewed all the questions. The reviewers relied on the symptoms and signs listed by the American College of Emergency Physicians [10] and their clinical judgment to determine whether or not it was an emergency or the need to go to the emergency department (ED) (Table 1). A total of 75 and 101 questions were determined to be an emergency and a non-emergency, respectively.

**Table 1:** Symptoms indicative of medical emergency in adults and pediatric population from the American College of Emergency Physicians (ACEP)

Adult medical emergency symptom	Pediatric medical emergency symptoms
Difficulty breathing and shortness of breath	Severe headache or vomiting, especially following a head injury
Chest or upper abdominal pain or pressure lasting 2 min or more	Abnormal or difficult breathing
Severe or persistent vomiting or diarrhea	Skin or lips that look blue, purple, or gray
Head or spine injury	Feeding or eating difficulties
Ingestion of a poisonous substance	Increasing or severe, persistent pain
Uncontrolled bleeding	Inability to stand up or unsteady walking
Coughing or vomiting blood	Unconsciousness
Suicidal or homicidal feelings	Seizures
Injury due to a serious motor vehicle accident, burns or smoke inhalation, near drowning, deep or large wound, or other serious injuries	Fever accompanied by a change in behavior (especially with a severe, sudden headache accompanied by mental changes, and neck/back stiffness)

These questions were input into each of the AI chatbots in a new chat per question to avoid memory retention bias, and the text outputs of ChatGPT, Google Bard, and Microsoft Bing AI Chat responses were evaluated (Table 2).

**Table 2:** Examples of Reddit questions and AI chatbots’ answers

Reddit questions	Reviewers	OpenAI’s ChatGPT	Google Bard	Microsoft Bing Chat
Today, I took my bottle of Xanax and all my bottles of gabapentin. Do I need medical attention for this overdose? My symptoms right now are just blurry vision and tiredness. Should I go to the ER?	Yes, this is an emergency	I'm really sorry to hear that you're going through this, but I can't provide the help that you need. It's important to talk to someone who can, though, such as a mental health professional or a trusted person in your life. They can offer support and guidance. If you're in the United States and in need of immediate help, consider reaching out to the National Suicide Prevention Lifeline at 1-800-273-TALK (1-800-273-8255) or seeking emergency medical attention by going to the nearest emergency room.	Yes, you should go to the ER. Xanax and gabapentin overdose can be dangerous, and you may need medical attention to prevent serious health problems or death. The symptoms of Xanax and gabapentin overdose can include blurred vision drowsiness, confusion, seizures, slow breathing, low blood pressure, and coma. If you are experiencing any of these symptoms, it is important to seek medical attention immediately. The ER can provide you with the care you need to prevent further health problems.	Yes, you should go to the emergency room immediately. Taking an overdose of Xanax and gabapentin can be dangerous and potentially life-threatening. It is important to seek medical attention as soon as possible to receive appropriate treatment. Please do not delay in seeking help. If you are unable to get to the emergency room on your own, call 911 or your local emergency number for assistance.
Is this an emergency or a normal constipation case? My stomach hasn't been feeling well since the last few days. There is gas all the time, and I have multiple urges to defecate, but nothing comes out.	No, this is not an emergency	I'm not a doctor, but based on the symptoms you've described, it may not necessarily be an emergency. However, it's always a good idea to consult a healthcare professional to get a proper evaluation and diagnosis. They can provide you with appropriate advice and guidance based on your specific situation.	In your case, since you have only been experiencing constipation for a few days, and it is not accompanied by any other symptoms, it is likely that it is a normal case of constipation. However, if your constipation does not improve after a few days, or if it gets worse, it is important to see a doctor to rule out any underlying medical conditions.	It's difficult for me to determine if this is an emergency or a normal case of constipation. However, I can provide you with some information on constipation that may help you. Constipation is generally described as having fewer than three bowel movements a week, passing lumpy or hard stools, straining to have bowel movements, feeling as though there's a blockage in your rectum that prevents bowel movements, or feeling as though you can't completely empty the stool from your rectum. Constipation may be considered chronic if you've experienced two or more of these symptoms for the last three months.

Because some of the AI chatbots’ answers were not explicit, a criterion was made in order to avoid ambiguity. The criterion describes three possible categories for the AI chatbot’s answer regarding whether or not it is considered an emergency or recommended to go to the ED: yes, no, or cannot determine. These were reviewed again by each of the authors based on the criteria previously established (Table 3).

**Table 3:** Study criteria to categorize AI chatbots’ responses

Yes	No	Cannot determine
Explicitly affirms that it is an emergency or that you should go to the ER/ED.	Explicitly affirms that it is not an emergency or that you should not go to the ER/ED.	When it states that it cannot determine if it is an emergency or not.
When it states that you should consider going to the ER without any other alternative.	When it states that it may not necessarily be an emergency.	When it cannot confidently determine whether or not it is an emergency and provides two scenarios to be determined by the user.
When it expresses an intention of urgency or immediacy of seeking medical attention.	When it does not express an intention of urgency or immediacy of seeking medical attention.	When it gives you two options: either go to the emergency room or contact a doctor, without stressing the need to go to the emergency room, making the user the one to make the decision.
When the first option that it tells is to go to the emergency. Other options must be clear on their secondary position.	When it refers you to a specialist.	When no other criteria are met.

ChatGPT free version was used, which runs OpenAI’s GPT-3.5. Google Bard was in its beta version at the time it was used. Microsoft Bing Chat, which uses OpenAI’s GPT-4, has three options available regarding its mode of conversation style: creative, balanced, and precise. Each time a question was typed into the chat, the precise option was selected. AI chatbots’ responses were compared with each other and with authors' responses. We compared the number of emergency cases (“yes” responses), non-emergency cases (“no” responses), and cases that could not be determined (“cannot determine” responses) between the three different chatbots and between reviewers and AI chatbots’ responses. For qualitative comparison between reviewers and AI chatbot responses, we considered the reviewer’s responses to be the standard of comparison for AI chatbots’ responses. Based on this, either of the two responses from reviewers was considered to be a “true” interpretation of the cases, designated as “true emergency” or “true non-emergency.” When AI chatbots’ responses (“yes” or “no”) were the same as reviewers’ responses (“yes” or “no”) in a case, it was considered as “true” emergency or “true” non-emergency, respectively. On the other hand, when comparing both responses, “false” emergency or “false” non-emergency was designated when AI chatbots’ responses differed from reviewers’ responses (Table 4). Finally, we compared the performance of each AI chatbot in emergency and non-emergency cases, and we performed Cochran’s Q test on RStudio statistical software to determine if there was a significant difference in their performance. We considered a p-value of <0.05. 

**Table 4:** Quantitative comparison between reviewers and AI chatbots’ responses

	Reviewers "yes" response	Reviewers "no" response
AI chatbots "yes" responses	True emergency	False emergency
AI chatbots "no" responses	False non-emergency	True non-emergency

## Results

The sample contained 176 questions/cases with the premise mentioned before. A total of 75 (43%) of these cases were considered as emergency by the reviewers, while the remaining 101 (57%) cases were considered as true non-emergency. 

Responses to these questions by the different AI chatbots were evaluated and categorized following the established criteria. OpenAI’s ChatGPT-3.5 considered 98 (56%) cases to be an emergency, while Google Bard and Microsoft Bing Chat considered 94 (53%) and 100 (57%) cases to be an emergency, respectively. Google Bard detected more true emergency cases compared to the other AI chatbots, with 65 (87%) out of the 75 true emergency cases, followed by Microsoft Bing and ChatGPT-3.5, with 61 (82%) and 58 (77%), respectively (Table 5). Nevertheless, none of the AI chatbots demonstrated to be superior to each other (p-value = 0.35) in detecting true emergency cases.

**Table 5:** Number of true and false emergency cases detected by the different AI chatbots None of the AI chatbots demonstrated to be superior to each other (p-value = 0.35) in detecting true emergency cases

Reviewers/AI chatbots	Number of emergency cases detected	Number of true emergency cases detected by AI chatbots	% of true emergency cases detected by AI chatbots	Number of false emergency cases interpreted by AI chatbots
Reviewers	75	N/A	N/A	N/A
OpenAI's ChatGPT-3.5	98	58	77%	40
Google Bard	94	65	87%	29
Microsoft Bing Chat	100	61	82%	39

ChatGPT-3.5 had the highest number of “false” emergency cases with 40 cases, followed by Microsoft Bing and Google Bard with 39 and 29 false emergency cases, respectively, as demonstrated in Table 5. 

The number of non-emergency cases from all cases detected by the different AI chatbots was the following: 38 (22%) for OpenAI ChatGPT, 36 (21%) for Google Bard, and 29 (16%) for Microsoft Bing. Out of the 101 true non-emergency cases considered by reviewers, ChatGPT-3.5 detected only 37 (36%) cases, while Google Bard detected 34 (33%) and Microsoft Bing detected 27 (26%). Once again, there was no significant difference (p-value = 0.16) between the different AI chatbots to detect non-emergency cases. Number of false non-emergency cases were two in both Google Bard and Microsoft Bing and only one in Google Bard (Table 6).

**Table 6:** Number of true and false non-emergency cases detected by AI chatbots There was no significant difference (p-value = 0.16) between the different AI chatbots to detect non-emergency cases

Reviewers/chatbots	Number of non-emergency cases detected	Number of true non-emergency cases detected by AI chatbots	% of true non-emergency cases detected by AI chatbots	Number of false non-emergency cases interpreted by AI chatbots
Reviewers	101	N/A	N/A	N/A
OpenAI's ChatGPT-3.5	38	37	36%	1
Google Bard	36	34	33%	2
Microsoft Bing Chat	29	27	26%	2

The different AI chatbots could not determine the cases as either emergency or non-emergency, in similar numbers. These cases eventually fell into the “cannot be determined” category. Out of the 176 cases, Google Bard and Microsoft Bing could not determine whether it was an emergency or not in 47 (27%) and 46 (26%), respectively, while ChatGPT had 40 (23%) undetermined cases.

## Discussion

The ED is a notably congested section within the hospital, catering to a diverse range of patients, encompassing high-risk individuals [11]. Overcrowding denotes a circumstance wherein the optimal functioning of the ED becomes compromised, primarily attributable to an excessive influx of patients awaiting consultation, diagnosis, treatment, transfer, or discharge [12].

Numerous factors contribute to this phenomenon. Among them, the volume of incoming patients plays a significant role, predominantly stemming from the inappropriate utilization of emergency services, such as unwarranted visits, non-urgent cases, and self-referred individuals [7].

In a broader context, excessive overcrowding leads to adverse repercussions on patient well-being, mortality rates, disease incidence, patient contentment, and the overall standard of healthcare provision [6,13,14]. Additionally, it gives rise to prolonged durations of stay within the ED, heightened instances of patients departing without receiving attention, and an elevated frequency of medical inaccuracies [15,16].

The emergence of urgent care clinics has arisen as a response to this issue, providing treatment for minor ailments or injuries that lack life-threatening severity, thereby distinguishing them from EDs designed for critical conditions [17]. Utilization of these facilities, rather than EDs, has witnessed a surge in recent times and has partially alleviated the problem of ED overcrowding to some extent [18,19], albeit remaining insufficient for complete resolution.

That being acknowledged, there still exists a critical necessity for looking into innovative solutions that can effectively differentiate genuine emergencies from situations that could be managed through alternative means. 

The most popular AI chatbots are OpenAI’s ChatGPT, Google Bard, and Microsoft Bing Chat. By January 2023, within a span of two months, ChatGPT garnered an approximate user base of 100 million, thereby achieving unparalleled rapid growth within the domain of web-based platforms [20]. Notably, Microsoft’s Bing strategically adopted ChatGPT, effectuating the inclusion of AI into its search mechanism. It is noteworthy that Google Bard, operating in a beta version, uses Google’s own model, called LaMDA, representing Google’s incursion into similar technological pursuits.

With the rising popularity of AI chatbots and their integration into healthcare [21,22], they stand as a promising strategy to ease the pressure on emergency services by efficiently assessing the urgency of situations and determining the need to seek emergency resources, thus ultimately contributing to more effective and resource-efficient emergency management systems.

In this cross-sectional study, the three different AI chatbots interpreted almost identical numbers of emergency, non-emergency, and undetermined cases. In general, AI chatbots considered around 12-15% more cases to be an emergency than reviewers. On the other hand, a very low number of cases were considered by AI chatbots to be non-emergency compared to reviewers (around 35% of cases less). A difference in numbers between each AI chatbot was noticeable when qualitatively comparing their responses to the reviewers. Google Bard detected the most true emergency cases (87%) and true non-emergency cases (36%). However, no real difference in performance between the three AI chatbots was found in detecting true emergencies (p-value = 0.35) and non-emergency cases (p-value = 0.16). 

Based on these findings, it can be asserted that further refinement is necessary for AI chatbots to discern between emergencies and non-emergencies accurately. Nonetheless, it should be noted that the original intention behind developing these AI chatbots did not encompass this objective. Consequently, it remains imperative to anticipate future advancements in AI technology dedicated to this precise task, accompanied by subsequent research efforts that substantiate these outcomes.

The implementation of AI chatbots specifically designed for differentiating between emergencies and non-emergencies might have the capacity to influence patient outcomes by promptly and accurately addressing patient inquiries, thereby potentially decreasing unnecessary clinical appointments and releasing valuable resources for those in greater need [23]. Additionally, the utilization of these AI chatbots could facilitate patient equity, catering to individuals with limited mobility, non-traditional work schedules, or concerns about medical expenses, thereby potentially fostering increased adoption of AI chatbot services [24].

Limitations

Chatbots cannot always give a direct answer to the main question of whether to go to an emergency room; in some cases, we observed that the response was not specified as it only gave vague information.

AIs are trained to generate answers by identifying specific words to understand the questions or inquiries. They obtain information through vast but not limitless resources like digital articles, books, and web pages. Those sources are predetermined by its creator when the AI is being developed, which means that it would not comprehend and make a needed answer with facts that are off its sources. Moreover, the constantly emerging medical discoveries become another limitation as they overcome the lack of automatically continuous updates for AI’s knowledge, restricting their source of information. 

Furthermore, chatbots are highly criticized for not being able to understand and differentiate human feelings and emotions [22]. AI processes the words in the questions literally, which can lead to underestimating true emergencies or assuming emergencies that indeed are exaggerations of the patient’s situation and that the AI cannot understand due to incorrect use of words in the description of the patient’s problem.

## Conclusions

In essence, the overcrowding issue within EDs arises due to a complex interplay of factors, leading to adverse effects on patient care and healthcare quality. Despite the emergence of urgent care clinics as a partial solution, effectively distinguishing genuine emergencies from manageable cases remains a central challenge.

AI chatbots like ChatGPT, Google Bard, and Microsoft Bing Chat require further refinement to accurately identify emergency situations. AI chatbots cannot be used at the moment to triage or understand medical emergencies. While originally not designed for this purpose, ongoing advancements in AI technology are expected to address this limitation, potentially revolutionizing emergency care and patient outcomes.

## References

[1] Caldarini G, Jaf S, McGarry K (2022). A literature survey of recent advances in chatbots. Information.

[2] Brandtzaeg PB, Følstad A (2017). Why people use chatbots. 2017 International Conference on Internet Science, Thessaloniki.

[3] Nadarzynski T, Miles O, Cowie A, Ridge D (2019). Acceptability of artificial intelligence (AI)-led chatbot services in healthcare: a mixed-methods study. Digit Health.

[4] Palanica A, Flaschner P, Thommandram A, Li M, Fossat Y (2019). Physicians' perceptions of chatbots in health care: cross-sectional web-based survey. J Med Internet Res.

[5] Ivanovic M, Semnic M (2018). The role of agent technologies in personalized medicine. 2018 5th International Conference on Systems and Informatics (ICSAI).

[6] Savioli G, Ceresa IF, Gri N (2022). Emergency department overcrowding: understanding the factors to find corresponding solutions. J Pers Med.

[7] Sartini M, Carbone A, Demartini A (2022). Overcrowding in emergency department: causes, consequences, and solutions-a narrative review. Healthcare (Basel).

[8] Nobles AL, Leas EC, Dredze M, Ayers JW (2020). Examining peer-to-peer and patient-provider interactions on a social media community facilitating ask the doctor services. Proc Int AAAI Conf Weblogs Soc Media.

[9] Ayers JW, Poliak A, Dredze M (2023). Comparing physician and artificial intelligence chatbot responses to patient questions posted to a public social media forum. JAMA Intern Med.

[10] (2023). Know when to go to the ER. https://www.emergencyphysicians.org/article/know-when-to-go/know-when-to-go-overview.

[11] Babatabar-Darzi H, Jafari-Iraqi I, Mahmoudi H, Ebadi A (2020). Overcrowding management and patient safety: an application of the stabilization Model. Iran J Nurs Midwifery Res.

[12] Lindner G, Woitok BK (2021). Emergency department overcrowding: analysis and strategies to manage an international phenomenon. Wien Klin Wochenschr.

[13] Badr S, Nyce A, Awan T, Cortes D, Mowdawalla C, Rachoin JS (2022). Measures of emergency department crowding, a systematic review. How to make sense of a long list. Open Access Emerg Med.

[14] Kenny JF, Chang BC, Hemmert KC (2020). Factors affecting emergency department crowding. Emerg Med Clin North Am.

[15] Carter EJ, Pouch SM, Larson EL (2014). The relationship between emergency department crowding and patient outcomes: a systematic review. J Nurs Scholarsh.

[16] Epstein SK, Huckins DS, Liu SW, Pallin DJ, Sullivan AF, Lipton RI, Camargo CA Jr (2012). Emergency department crowding and risk of preventable medical errors. Intern Emerg Med.

[17] Graham King MD (2023). Graham King MD. Emergency vs. urgent care: differences. Mayo Clinic Health System. https://www.mayoclinichealthsystem.org/hometown-health/speaking-of-health/emergency-vs-urgent-care-whats-the-difference.

[18] Black LI, Adjaye-Gbewonyo D (2021). Urgent care center and retail health clinic utilization among adults: United States. NCHS Data Brief.

[19] Hong AS, Froehlich T, Clayton Hobbs S, Lee SJ, Halm EA (2019). Impact of a cancer urgent care clinic on regional emergency department visits. J Oncol Pract.

[20] (2023). UBS. Let’s chat about ChatGPT. https://www.ubs.com/us/en/wealth-management/insights/market-news/article.1585717.html.

[21] Jiang F, Jiang Y, Zhi H (2017). Artificial intelligence in healthcare: past, present and future. Stroke Vasc Neurol.

[22] He J, Baxter SL, Xu J, Xu J, Zhou X, Zhang K (2019). The practical implementation of artificial intelligence technologies in medicine. Nat Med.

[23] Rasu RS, Bawa WA, Suminski R, Snella K, Warady B (2015). Health literacy impact on national healthcare utilization and expenditure. Int J Health Policy Manag.

[24] Herzer KR, Pronovost PJ (2021). Ensuring quality in the era of virtual care. JAMA.

